# Clinical impact of medication reviews for community-dwelling patients in primary healthcare

**DOI:** 10.1186/s12875-023-02216-0

**Published:** 2023-12-02

**Authors:** Annika Dobszai, Cecilia Lenander, Beata Borgström Bolmsjö, Katarina Wickman, Sara Modig

**Affiliations:** 1 Center for Primary Health Care Research, Department of Clinical Sciences Malmö, Lund University, Malmö, Sweden; 2 Primary Health Care Skåne County, Lund, Sweden; 3 Department of Medicines Management and Informatics in Skåne County, Malmö, Sweden

**Keywords:** Clinical relevance, Drug-related problem, Independent living, Medication review, Pharmacist, clinical, Polypharmacy, Primary healthcare

## Abstract

**Background:**

A high number of drug-related problems has previously been shown among community-dwelling patients in primary healthcare in Skåne County, Sweden. Medication reviews are one way to solve these problems, but their impact is largely dependent on the process. We aimed to evaluate medication reviews for community-dwelling patients regarding the clinical relevance of the pharmacists’ recommendations, and their implementation by general practitioners. We also wanted to investigate if the general practitioners’ tendency to act on drug-related problems was correlated to different factors of the process.

**Methods:**

This was a cohort study, where patients in primary healthcare considered in need of a medication review were selected. Pharmacists identified drug-related problems and gave written recommendations on how to solve the problems to the general practitioner, via the medical record, and in addition in some cases via verbal communication. The clinical relevance of the recommendations was graded according to the Hatoum scale, ranging from one (adverse significance) to six (extremely significant). Descriptive statistics were used regarding the clinical relevance and the general practitioners´ tendency to act on drug-related problems. Multiple logistic regression analysis was used to examine the association between the tendency to act and different factors of the process.

**Results:**

A total of 96.1% of the 384 assessed recommendations from the pharmacists were graded as significant or more for the patient (Hatoum grade 3 or higher). The general practitioners acted on 63.8% of the drug-related problems. Fewer recommendations per patient, as well as verbal communication in addition to written contact, significantly increased the general practitioners’ tendency to act on a drug-related problem. No significant association was seen between the tendency to act and the clinical relevance of the recommendation.

**Conclusions:**

The high proportion of clinically relevant recommendations from the pharmacists in this study strengthens medication reviews as an important tool for reducing drug-related problems. Verbal communication between the pharmacist and the general practitioner is important for measures to be taken. Multiple recommendations for the same patient reduced their likelihood to of being addressed by the general practitioner.

## Background

The treatment of elderly people is, in many cases a challenge, due to polypharmacy that often comes along with multiple chronic diseases [1]. Besides costs for unplanned hospitalization, drug-related problems (DRPs) often lead to inconveniences for the patient [2]. Many times, DRPs can be avoided by adjusting the treatment based on the individual patient´s unique conditions, benefits, and risks. Medication reviews (MRs) can contribute to the effort to prevent and reduce DRPs [3, 4].

In Skåne County in the south of Sweden, MRs are primarily conducted for hospitalized patients and for patients living in nursing homes. The MRs in nursing homes follow an elaborated structure with pharmacists, physicians and nurses cooperating with the process [5–9].The nurse documents the patient’s symptoms according to a validated assessment tool. The pharmacist identifies potential DRPs and gives suggestions to the physician on how to solve them. A subsequent team discussion supports the physician’s decision-making. The aim is to achieve higher quality and safety in the patient’s medication treatment. For community-dwelling patients in primary healthcare, MRs are not as common as in nursing homes but are on the rise. Data concerning MRs for this new target group are relatively scarce from the Nordic countries. International studies exists, but with varying settings and procedures [10–13]. Our previously published data from community-dwelling patients [14] showed a higher number of DRPs compared to Swedish studies conducted in nursing homes [8, 9], especially cases of renal impairment or polypharmacy. However, the impact of an MR on the quality and safety of a patient’s treatment is largely dependent on the process. It is therefore important to assess the clinical relevance and the implementation of the recommendations given by the pharmacists. This process has not been evaluated previously for community-dwelling patients in primary healthcare.

We aimed to evaluate MRs for community-dwelling patients regarding the clinical relevance of the pharmacists’ recommendations, and the implementation of the recommendations by the general practitioners (GPs). We also wanted to investigate if the GPs’ tendency to act on the DRP was correlated to different factors in the process.

## Methods

### Study design and participants

This was a non-controlled cohort study. GPs and nurses at 14 public primary healthcare centers participated, as well as 15 clinical pharmacists in Skåne County. MRs for community-dwelling patients was conducted as a newly introduced part of routine healthcare. In their daily work the GPs and nurses at the healthcare centers selected community-dwelling patients in need of an MR due to, for example, suspected DRPs. There were no further inclusion criteria regarding for example number of medications. An informed consent was collected from all included patients. A total of 165 MRs were conducted according to the model and 109 patients were included in the study. Patients living in nursing homes, younger than 18 years, or with protected identity were not included. Ethics approval was granted, and all methods were carried out in accordance with relevant guidelines and regulations.

### Procedure

The MRs were conducted from the third quarter of 2018 until the fourth quarter of 2020, in the southern part of Skåne County. The MRs were performed according to a version of the Lund Integrated Medicine Management (LIMM). LIMM is originally a structured model for MRs for hospitalized patients, developed in southern Sweden, where pharmacists identify, and within multi-professional teams solve DRPs [5, 6]. The model has been adjusted to suit primary healthcare [9]. The patients answered a symptom scale, PHASE-20 (PHArmacotherapeutical Symptom Evaluation, 20 questions) [15], which was then sent to the clinical pharmacists together with a current medication list. PHASE-20 is a validated assessment tool for identifying symptoms that may be caused by medication treatment. The tool is based on the presence of symptoms from 19 groups, and one additional open question. PHASE-20 is used for MRs in most counties in Sweden. Based on the received PHASE-20, the current medication list and information from the electronic medical record, the clinical pharmacists identified potential DRPs among the patients. In Sweden electronic medical records is used for all patients. The pharmacists compiled the identified DRPs in the electronic medical record, together with recommendations to the GPs on how to solve the DRPs. Two of the clinical pharmacists, with solid experience, who were also part of the research group, categorized the recommendations into ten categories for the analysis. In case of discrepancy or difficulty in the classification, the pharmacists reached consensus through discussion. The categories followed the existing classification from the template in the electronic medical record, with an addition of the category *Consider other measure*, and consisted of:

1. For information/notification
2. Consider initiation of drug therapy
3. Consider withdrawal of drug therapy
4. Consider reduced dose
5. Consider increased dose
6. Consider dose regimen adjustment
7. Consider change in drug formulation
8. Consider change of drug therapy
9. Consider evaluation of drug therapy
10. Consider other measure

In addition to the compilation in the medical record, the pharmacist and the GP, in some cases, discussed the recommendations by phone or in a digital meeting. The GPs decided on appropriate measures. Further information about the procedure is presented in previously published work [14].

### Data collection

The extent of the GPs’ implementation of the pharmacists’ recommendations within two months after receiving the recommendations was retrieved from the electronic medical record. The time limit of two months was chosen according to the process used in previous similar studies [8, 9]. The information was compiled and categorized by one of the researchers according to *Implemented/Partly implemented/Planned/Measures other than proposed taken/No action taken*. The research group reviewed a number of samples to ensure a rigorous process. Cases where the GP intended to take a proposed measure, but did not due to the patient’s objection, was categorized as *No action taken*, considering the intention of this study was to assess the actual implementation. If no information could be found in the medical record, the category *No action taken* was chosen. The categories *Implemented*, *Partly implemented*, *Planned,* and *Measures other than proposed taken* were also merged and presented as *Action taken*.

The collected information from the MRs was used to assess the clinical relevance of the recommendations from the pharmacists. The collected data was sent to two physicians, with no clinical connection to the included patients, and with solid competence and experience in medication safety issues i.e., one geriatrician and the other a GP responsible for the area medication safety for the elderly in the county. The physicians assessed the recommendations independently using the Hatoum’s ranking scale [16]. There is no standardized method for this evaluation, but ranking according to Hatoum et al. has previously been applied in a few Swedish studies [17, 18]. The Hatoum’s ranking scale consists of six rating steps, as shown in Table 1.

**Table 1:** Description of ranking steps of clinical relevance according to Hatoum et al

Ranking	Label	Interpretation
1	Adverse significance	Recommendation supplied by the clinical pharmacist may lead to adverse outcome
2	No significance	Recommendation is informational (not specifically related or meaningful to the patient in question)
3	Somewhat significant	Benefit of the recommendation to the patient could be neutral depending on professional interpretation
4	Significant	Recommendation would bring care to a more acceptable and appropriate level (i.e., standards of practice)
5	Very significant	Recommendation qualified by a potential or existing major organ dysfunction
6	Extremely significant	Information qualified as a life and death situation

In cases with different judgments between the two physicians regarding the ranking, in a second step, they reached consensus through discussion.

### Data analysis

The number of recommendations per patient was compiled, as was the number of MRs where the pharmacist and the GP had both verbal and written contact, compared to written only. Descriptive analysis was used for the ranking of clinical relevance of the recommendations from the pharmacists. The extent of agreement between the two physicians was measured with weighted Cohen´s kappa. Descriptive statistics were used regarding the GPs’ tendency to act on the recommendations from the pharmacist. Furthermore, logistic regression analysis was used to examine the association between the GPs’ tendency to act and the degree of clinical relevance, the number of recommendations per patient, and a verbal contact or not between the pharmacist and the GP about the recommendations in addition to written contact, respectively. In the regression analysis the above-described categories *Action taken,* and *No action taken* were used as dependent variables regarding the implementation. Data was analyzed using IBM SPSS version 28. A *p*-value < 0.05 was considered statistically significant.

## Results

Of the 109 included patients, a total of 60 (55%) were women and the median age was 79 years with a range of 52–98 years. The median number of medications per patient was 11 (range 5–28). The clinical pharmacists identified 420 potential DRPs among the patients, giving a mean of 3.9 DRPs per patient and a median of 4 DRPs per patient (range 0–13), as shown in our previous study [14]. Based on the identified DRPs, the pharmacists gave 420 recommendations regarding the drug treatment. The most frequent types of recommendations were *Consider withdrawal of drug therapy* (*n* = 96, 22.9%), *Consider evaluation of drug therapy* (*n* = 66, 15.7%) and *Consider reduced dose* (*n* = 57, 13.6%). The mean (SD) number of recommendations per patient was 3.5 (2.7) and the median number was 4 (range 0–12). The pharmacists and the GPs had verbal contact, in addition to written contact only, regarding 49 (45.0%) of the MRs.

**Fig. 1:**
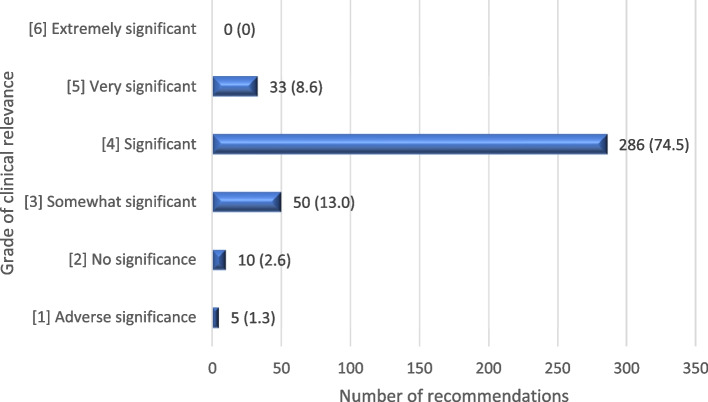
Clinical relevance of recommendations, graded according to Hatoum et al., n = number of recommendations (%). *N* = 384

The clinical relevance was ranked for 384 of the recommendations from the pharmacists to the GPs. The category *For information/notification* was excluded from the ranking, as was recommendations from the pharmacist given directly to the patient. The recommendations directly to the patient could concern, for example, information about compliance or problems regarding the therapy instructions. Of the 384 ranked recommendations, 96.1% were graded as three or higher according to Hatoum’s ranking scale, and 83.1% were graded as four or higher, as shown in Fig. 1. Five of the recommendations (1.3%) were graded as one, *Adverse significance.* These suggestions were merely recommendations according to clinical guidelines, such as prescribing GLP1-agonist to a frail patient with multimorbidity. The recommendations were however considered not to risk any harm if implemented.

The weighted Cohen’s kappa was 0.63, indicating substantial agreement between the two physicians (Table 2). In 82.3% of the cases, the physicians were in complete agreement. The non-consistent gradings differed by one grade in 65 cases, two grades in two cases, and three grades in just one case. Consensus discussions led to the higher grade in 37 (54.4%) cases.

**Table 2:** Distribution of ranking of clinical relevance according to Hatoum et al., between the two physicians

				**Physician 2**			
	**Ranking**	**1**	**2**	**3**	**4**	**5**	**6**
	**1**	3		1	1		
	**2**		8	2			
**Physician 1**	**3**			31	33	1	
	**4**			6	258	5	
	**5**				19	16	
	**6**						

The GPs acted on 245 (63.8%) of the 384 analyzed recommendations. For 139 recommendations (36.2%), no action was taken. Out of these, information about any action could not be found in the electronic medical record regarding 43 recommendations (11.2%). The distribution into the described categories were: *Implemented* 163 (42.4%); *Partly implemented* 20 (5.2%); *Planned* 34 (8.9%); *Measures other than proposed taken* 28 (7.3%); *No action taken* 139 (36.2%).

The results from the multivariable logistic regression analysis are presented in Table 3. The odds for the GPs to act on a DRP, with following recommendation, was 2.4 times higher after written and verbal communication with the pharmacist, compared to written contact only (OR 2.4, 95% CI 1.52–3.79 and *p*-value < 0.001). The odds for the GPs acting on a DRP was 0.58 when the pharmacist gave five or more recommendations/patient, compared to 1–4 recommendations/patient (OR 0.58, 95% CI 0.37–0.91 and *p*-value 0.016). The division into 1–4 and ≥ 5 recommendations/patient was made with regard to equal distribution in the group as well as clinical plausibility. No significant association was seen between the GPs’ tendency to act and the degree of clinical relevance of the recommendation.

**Table 3:** Logistic regression analysis of different factors in the process, with the dichotomous outcome GPs tendency to act on DRPs

	Model 1 (univariable)		Model 2 (multivariable)	
**Variable**	**OR (95% CI)**	***p*-value**	**OR (95% CI)**	***p*-value**
Verbal and written communication (reference: written only)	2.52 (1.60–3.96)	** < 0.001**	2.40 (1.52–3.79)	** < 0.001**
≥ 5 recommendations/patient (reference: 1–4 recommendations)	0.54 (0.35–0.83)	**0.005**	0.58 (0.37–0.91)	**0.016**
Graded clinical relevance of the recommendation (1–6, ordinal scale)	1.06 (0.77–1.46)	0.72	1.01 (0.72–1.42)	0.946

Model 1: Separate univariable logistic regression of each variable

Model 2: Multiple logistic regression including the three presented variables

## Discussion

As much as 96% of the recommendations made by the clinical pharmacists to the GPs regarding identified DRPs were graded to be clinically relevant. The result strengthens MRs as an important tool to reduce DRPs among community-dwelling patients in primary healthcare. The GPs acted on 64% of the DRPs with following recommendations. Fewer recommendations per patient, as well as verbal communication in addition to written contact, increased the GPs’ tendency to act. No correlation was seen between the tendency to act and the degree of clinical relevance of the recommendation.

Given the high clinical relevance of the recommendations from the pharmacists in our study, one may wonder why not more measures were taken. In some cases, there was no sign in the electronic medical record that the GP had read or considered the recommendations, which might have affected the outcome. Possible explanations might be deficiencies in the model, such as lack of time for the GPs to process the information, misunderstanding of the process, need for education, or just a matter of different opinions. Furthermore, the model with MRs for community-dwelling patients was new to the participants, and new processes may need time to implement.

In previous studies, the acceptance rate of pharmacists’ recommendations varied hugely between 30–87%, albeit in different settings [2]. A wide spread of factors is discussed to influence the results/acceptance rate, such as the number of medications, diagnoses, and geographic differences [19–21]. One aspect to consider is the clinical reasoning and final assessment of the individual treatment that is made by the GP. The pharmacists highlight changes and evaluations to consider, facilitating the GPs’ decision-making, with the mutual aim of improving quality of treatment and well-being for the patient. Nevertheless, prescribing and deprescribing for elderly patients is a complex process. An acceptance rate of 100% is likely not appropriate to strive for. Furthermore, in some cases, no action was taken due to the patient’s reluctance to change treatment.

We saw no association between the grade of clinical relevance of the pharmacists’ recommendations and whether or not action was taken by the GPs. One reason for this could be that a recommendation with higher clinical relevance might be more challenging to implement. For instance, an adjustment of vitamin B-treatment is easy to apply, but of little importance to the patient, compared to discontinuation of a benzodiazepine, which is more demanding but could increase patient safety. Monzon-Kenneke et al. implied in their work that additional deprescribing would have occurred if the pharmacist was readily available to provide step-by-step instructions [13]. The most frequent type of recommendation from the pharmacists in this study, *Consider withdrawal of drug therapy*, may thus not always be uncomplicated to manage.

The odds for the GP to act on a DRP were significantly higher after verbal communication with the pharmacist, compared to written contact only. Verbal contact might give an opportunity to discuss details and supplementary questions and is likely to facilitate changes in the treatment regimen. An additional effect is the component of mutual learning among the participants. The results are in accordance with the idea of team-based care and “Good quality, local health care”, promoted by the Swedish government and the National Board of Health and Welfare [22, 23]. Communication is often facilitated through already-established collaborations. In addition, the verbal contact provides a guarantee that the GP becomes aware of the recommendations from the pharmacist, which was not always obvious in this study, based on information collected from the medical record. Some prior Swedish studies with a higher tendency to act (acceptance rate) were performed solely using face-to-face discussions (Lenander 2018; 80%, Bondesson 2012; 90%, compared to 64% in this study) [8, 17]. These previous results strengthen our finding that the tendency to act increases with verbal communication.

Multiple recommendations for the same patient significantly reduced the likelihood of addressment by the GP. The result is of value addressing the further performance of MRs following a similar model. Although each individual recommendation might be clinically relevant, it is important to take patient safety into account and thus not take too many measures at the same time. Elderly patients are often more fragile and changes in treatment must be made with caution [24, 25]. In this study, the patients were living independently and, in many cases, handled their own medications. A GP might be more restrained regarding major adjustments in treatment when the patient has less supervision from healthcare personnel for follow-up. In addition, too many alterations may be confusing for the patient and potentially lead to misunderstandings and new errors. In a nursing home, more structured monitoring is possible, thus making changes easier to implement. Nevertheless, patients living independently are an important target group for MRs, although they may need to be handled more carefully. The results also confirm that it may be wise for pharmacists to limit and prioritize more strictly regarding the number of recommendations.

This study has some limitations. The pharmacists conducting the MRs may have been extra thorough in their work since they knew they were part of a study. This might have led to a higher number of DRPs and following recommendations per MR. However, a vast majority of the recommendations were ranked as significant to the patient, which contradicts the likelihood of this effect. It is also possible that a larger number of included patients may have affected the results regarding possible association between the clinical relevance and the implementation of recommendations. Furthermore, no additional variables were used in the regression analysis such as patient or physician characteristics. Another weakness is the variation of documentation by the GPs in the medical record, which means that information on possibly implemented measures could not always be found. Nor could the GPs’ reasons for not acting on a DRP be retrieved or evaluated. In a future study, it may be interesting to explore the GPs’ decision-making process. A strength of this study is that, to our knowledge, it is the first to evaluate the process of MRs for community-dwelling patients in primary healthcare in terms of clinical relevance of recommendations and different aspects affecting implementation. The use of trained clinical pharmacists, and a well-established model for the MRs (a modified version of LIMM), ensures the consistency and reproducibility of the work. Another strength is that one of the two physicians conducting the ranking of the pharmacists’ recommendations was an experienced physician brought in from outside the research group, to enable independent estimates and ensure that assessment bias was not at risk. In future studies, it would be interesting to evaluate the clinical impact of the MRs for community-dwelling patients on hard endpoints such as hospital admissions, falls or quality of life-measures.

## Conclusions

The high proportion of clinically relevant recommendations from pharmacists emphasizes the importance of MRs to avoid DRPs among community-dwelling patients in primary healthcare. The odds for the GP to act on a DRP were significantly higher after verbal communication between the GP and the pharmacist, and with fewer recommendations per patient. This is important knowledge to incorporate when planning for the implementation of MRs for community-dwelling patients.

## Data Availability

The datasets used and analyzed during the current study are available from the corresponding author upon reasonable request.

## Abbreviations

DRP: Drug-Related Problem
GP: General Practitioner
LIMM: Lund Integrated Medicines Management
MR: Medication Review
PHASE-20: PHArmacotherapeutical Symptom Evaluation, 20 questions
